# The TeleKidSeq pilot study: incorporating telehealth into clinical care of children from diverse backgrounds undergoing whole genome sequencing

**DOI:** 10.1186/s40814-023-01259-5

**Published:** 2023-03-22

**Authors:** Monisha Sebastin, Jacqueline A. Odgis, Sabrina A. Suckiel, Katherine E. Bonini, Miranda Di Biase, Kaitlyn Brown, Priya Marathe, Nicole R. Kelly, Michelle A. Ramos, Jessica E. Rodriguez, Karla López Aguiñiga, Jessenia Lopez, Estefany Maria, Michelle A. Rodriguez, Nicole M. Yelton, Charlotte Cunningham-Rundles, Katie Gallagher, Thomas V. McDonald, Patricia E. McGoldrick, Mimsie Robinson, Arye Rubinstein, Lisa H. Shulman, Steven M. Wolf, Elissa Yozawitz, Randi E. Zinberg, Noura S. Abul-Husn, Laurie J. Bauman, George A. Diaz, Bart S. Ferket, John M. Greally, Vaidehi Jobanputra, Bruce D. Gelb, Carol R. Horowitz, Eimear E. Kenny, Melissa P. Wasserstein

**Affiliations:** 1 Department of Pediatrics, Division of Pediatric Genetic Medicine, Children’s Hospital at Montefiore/Montefiore Medical Center/Albert Einstein College of Medicine, 3411 Wayne Ave, 9th Floor, Bronx, NY 10467 USA; 2 The Institute for Genomic Health, Icahn School of Medicine at Mount Sinai, New York, NY USA; 3 Department of Medicine, Icahn School of Medicine at Mount Sinai, New York, NY USA; 4 Department of Population Health Science and Policy, Icahn School of Medicine at Mount Sinai, New York, NY USA; 5 Institute for Health Equity Research, Icahn School of Medicine at Mount Sinai, New York, NY USA; 6 Department of Pediatrics, Icahn School of Medicine at Mount Sinai, New York, NY USA; 7 Department of Medicine (Cardiology), Montefiore/Montefiore Medical Center/Albert Einstein College of Medicine, Bronx, NY USA; 8 Department of Pediatrics, Division of Child Neurology, New York Medical College, Valhalla, NY USA; 9 Pediatric Neurology, Boston Children’s Health Physicians/Maria Fareri Children’s Hospital, Hawthorne, NY USA; 10 Bethel Gospel Assembly, New York, NY USA; 11 Department of Allergy and Immunology, Children’s Hospital at Montefiore/Montefiore Medical Center/Albert Einstein College of Medicine, Bronx, NY USA; 12 Department of Pediatrics, Division of Developmental Medicine, Rose F. Kennedy Children’s Evaluation & Rehabilitation Center at Children’s Hospital at Montefiore/Montefiore Medical Center/Albert Einstein College of Medicine, Bronx, NY USA; 13 Isabelle Rapin Division of Child Neurology of the Saul R Korey Department of Neurology at Montefiore Medical Center/Albert Einstein College of Medicine, Bronx, NY USA; 14 Department of Pediatrics, Children’s Hospital at Montefiore/Montefiore Medical Center/Albert Einstein College of Medicine, Bronx, NY USA; 15 Department of Genetics and Genomic Sciences , Icahn School of Medicine at Mount Sinai, New York, NY USA; 16 Department of Obstetrics, Gynecology and Reproductive Science, Icahn School of Medicine at Mount Sinai, New York, NY USA; 17 Department of Pediatrics, Division of Ambulatory Pediatrics, Albert Einstein College of Medicine, Bronx, NY USA; 18 Molecular Diagnostics, New York Genome Center, New York, NY USA; 19 Department of Pathology and Cell Biology, Columbia University Medical Center, New York, NY USA; 20 Mindich Child Health and Development Institute, Icahn School of Medicine at Mount Sinai, New York, NY USA

**Keywords:** Whole genome sequencing, Genomic sequencing, Telehealth, Telegenetics, Genetic counseling, Clinical utility, Healthcare utilization, Pediatric genetics, Underrepresented populations

## Abstract

**Background:**

The COVID-19 pandemic forced healthcare institutions and many clinical research programs to adopt telehealth modalities in order to mitigate viral spread. With the expanded use of telehealth, there is the potential to increase access to genomic medicine to medically underserved populations, yet little is known about how best to communicate genomic results via telehealth while also ensuring equitable access. NYCKidSeq, a multi-institutional clinical genomics research program in New York City, launched the TeleKidSeq pilot study to assess alternative forms of genomic communication and telehealth service delivery models with families from medically underserved populations.

**Methods:**

We aim to enroll 496 participants between 0 and 21 years old to receive clinical genome sequencing. These individuals have a neurologic, cardiovascular, and/or immunologic disease. Participants will be English- or Spanish-speaking and predominantly from underrepresented groups who receive care in the New York metropolitan area. Prior to enrollment, participants will be randomized to either genetic counseling via videoconferencing with screen-sharing or genetic counseling via videoconferencing without screen-sharing. Using surveys administered at baseline, results disclosure, and 6-months post-results disclosure, we will evaluate the impact of the use of screen-sharing on participant understanding, satisfaction, and uptake of medical recommendations, as well as the psychological and socioeconomic implications of obtaining genome sequencing. Clinical utility, cost, and diagnostic yield of genome sequencing will also be assessed.

**Discussion:**

The TeleKidSeq pilot study will contribute to innovations in communicating genomic test results to diverse populations through telehealth technology. In conjunction with NYCKidSeq, this work will inform best practices for the implementation of genomic medicine in diverse, English- and Spanish-speaking populations.

**Supplementary Information:**

The online version contains supplementary material available at 10.1186/s40814-023-01259-5.

## Background

NYCKidSeq is a multi-institutional, clinical genomics research program in New York City (NYC). It is one of six research programs funded as part of the Clinical Sequencing Evidence-Generating Research (CSER2) consortium, jointly funded by the National Human Genome Research Institute and the National Institute on Minority Health and Health Disparities [1]. NYCKidSeq is focused on developing and testing strategies for enhancing communication of genomic information in health systems and evaluating the utility of advanced genomic sequencing technology for improving diagnostic rates in populations representative of the rich diversity of NYC [2]. With the continued threat of COVID-19, healthcare centers and research programs have adapted their service delivery models to protect patients and providers from possible exposure [3]. When the NYCKidSeq clinical trial was interrupted by institutionally required changes to in-person patient care, we recognized the opportunity to evaluate alternative forms of genomic communication with families from underserved populations. TeleKidSeq is a pilot study emerging from the NYCKidSeq program that was developed to examine the impact of innovative remote genetic counseling modalities in medically underserved populations who historically have had limited access to genetics services.

Although in-person genetic counseling was the most common service delivery model before 2020 [4], telehealth (defined broadly as the delivery of healthcare services via telephone or videoconferencing technology) has been utilized and studied for over a decade across various genetics specialties and in remote, mostly rural settings [5–7]. It has been previously shown that patient satisfaction, knowledge of genetics, and psychological outcomes associated with remote genetic counseling services are on par with those of in-person counseling. In addition, telehealth in genetics practice has been shown to reduce travel time and costs for patients [5, 7–9]. Numerous centers offering genetic counseling have described their experiences in pivoting to either fully remote or hybrid (incorporating both remote and in-person components) models of service during the COVID-19 pandemic. These centers have detailed their specific operational changes and have generated additional evidence and support for the feasibility of telehealth in genetics clinical practice and its acceptability among patients and genetics professionals [10–14].

Although telehealth holds promise for increasing access to genetics services, it is critical to acknowledge that not all patient communities have equitable access to the technology required for virtual visits. Previously cited challenges to successful implementation of telehealth in clinical genetics include insufficient internet access and connectivity to support a virtual session; lack of physical privacy, hampering patient confidentiality; distraction by children and other members of the household; lack of knowledge and comfort using new technology; language and literacy barriers limiting the ability to connect to telehealth platforms or complete genetic testing forms; and complexities of billing and reimbursement [15–19]. As the majority of studies of remote genetics service delivery have been conducted among European descent, English speaking participants [16], the field must assess the information needs and preferences of racially, ethnically, and socioeconomically diverse patients.

Due to the increased demand for genetic counseling, the evaluation of different telehealth modalities [20], including study of specific capabilities and features of telehealth platforms, has been warranted and ongoing. Previous studies have shown that the use of visual aids in genetic counseling improves patients’ understanding of complex genetic concepts [21–23], although these studies were performed in more traditional in-person settings. To date, no study has explored how using visual aids via screen-sharing in telehealth visits influences patient understanding and retention of the information discussed. We hypothesize that sharing visual representations of the discussed genetic information in real-time (screen-sharing) during telehealth genetic counseling visits will enable better patient education and understanding of results and follow-up recommendations as compared to telehealth without screen-sharing capabilities. The TeleKidSeq pilot study will assess the impact of videoconferencing screen-sharing capabilities on families who are undergoing genomic testing through the NYCKidSeq program. The pilot study aims to recruit 496 participants with suspected genetic disorders. This consists of children 0 to 17 years and young adults 19 to 21 years from racially and ethnically diverse and medically underserved communities of NYC. Participants will be randomized to one of two study arms: videoconferencing with screen sharing (ScrS) and videoconferencing without screen-sharing (NScrS) for the genetic counseling results disclosure appointment. We will then assess parental understanding, satisfaction, and feelings about the results, and their subsequent behaviors. This study will also evaluate the utility of genome sequencing for increased diagnosis of study participants with neurologic, cardiovascular, and immunologic conditions.

## Methods/design

### Study design overview

**Fig. 1:**
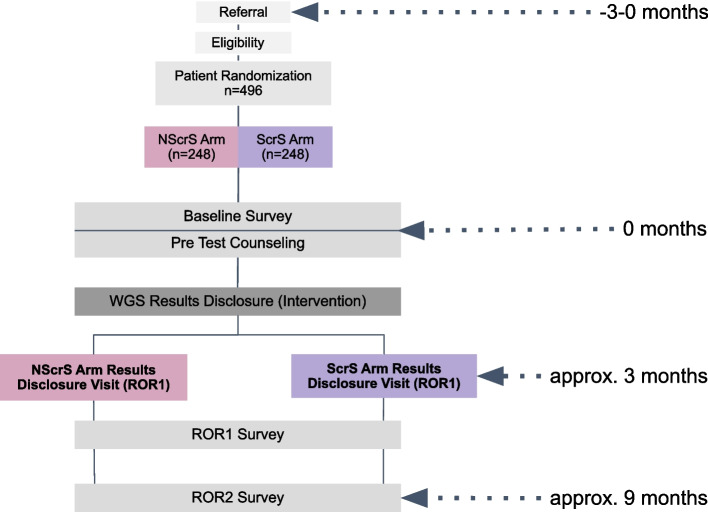
**TeleKidSeq study design**. Participants are randomized to the no screen-share (NScrS) and screen-share (ScrS) arm. Participants in both arms receive genome sequencing and complete surveys at baseline, after result disclosure (ROR1 survey), and 6 months after result disclosure (ROR2 survey). Results disclosure of participants in the NScrS arm (*n* = 248) is conducted without the use of screen-sharing and any relevant images are held up to the camera. Results disclosure of participants in the ScrS arm (*n* = 248) is conducted with the use of screen-sharing

Figure 1 represents the TeleKidSeq study design. Participants will be randomized based on study institution and primary phenotypic indication to either the return of genomic results using videoconferencing with or without screen-sharing. Both arms will use our Genomic Understanding, Information and Awareness (GUÍA) genomic result disclosure tool in real time (screen-share) and/or sent after the completed visit. GUÍA is a novel digital application that facilitates delivery of individualized genomic results and clinical information in a personalized, highly visual, and narrative manner, and has been described previously [24]. Genome sequencing will be performed for each participant, either singleton, duo, or trio (depending on sample availability). Surveys administered at baseline (BL, 0 months), results disclosure (ROR1, approximately + 3 months), and 6-month follow-up (ROR2, approximately + 9 months) will collect participant outcomes. Results disclosure is estimated to occur at approximately 3 months. This 3-month estimate was established based on the expected time to receive and reconcile clinical reports as well as the time required by the genetic counselor and the referring provider to review, clinically interpret, and determine the appropriate recommendations for the proband and family. All CSER member-sites will be required to administer a post-return of results survey 5–7 months after disclosure of genomic results. CSER consortium investigators selected an interval of 5–7 months for the ROR2 survey as it was most feasible across all CSER sites, could fit within the constraints of the funded study period, and was likely to capture most of the impact of the result. As a member-site of the CSER consortium, the funding source has a role in the design of this study with regard to its recruitment goals and outcome measures [1].

### Recruitment, enrollment, and sample size

The TeleKidSeq study aims to recruit 496 participants, with > 60% representing racially and ethnically diverse populations [25]. The target enrollment of 496 participants is based on the remainder for NYCKidSeq target enrollment (634 participants were enrolled in NYCKidSeq at its completion) and funding availability. Individuals will be considered underrepresented or medically underserved based on race/ethnicity as well as the U.S. Health Resources & Services (HRSA) definition of medically underserved status, which will be determined by participant home address [26]. This definition of medically underserved/underrepresented populations will be employed across all CSER sites [1, 26]. Referring providers will be educated by the study team on eligibility criteria and how to send referrals to the study team. To initiate a referral, referring providers must complete a “phenotype checklist” for each patient, which specifies the patient’s primary indication for genome sequencing and other relevant clinical features. Referring physicians must first introduce the study to the parents/guardians of the patient (described as proband throughout) during a routine clinic visit, phone call, or inpatient admission. Upon receipt of a completed phenotype checklist, the study staff will assess patients’ eligibility, technological literacy, and access to videoconferencing equipment (i.e., availability of an electronic device with video capability, stable wireless connectivity, and a private location to complete the visit) using a telehealth screener survey. For participants with barriers to telehealth identified either during administration of telehealth screener or during a visit, study staff will offer to coordinate a visit at a study site, where participants can use the study provided technology to complete their visits with a study GC. For those who are able to conduct a virtual visit, a BL visit via videoconferencing will be scheduled. The location of participants during the visit, device used, and occurrence of any connectivity issues are captured in the study record, which will be used for analysis. Participants enrolled will receive $80 total in gift cards for completing all three study visits (BL, ROR1, and ROR2).

### Inclusion and exclusion criteria

Potential participants will be aged 0–21 years, have a suspected genetic cause for their neurologic, cardiac, or immunologic condition, and receive care from a physician at a participating academic medical center or a partner external center within the New York metropolitan area. Parents or legal guardians of the patients must be English- or Spanish-speaking. Probands of any cognitive ability are eligible. Probands ages 18–21 who are cognitively intact must have a parent/guardian willing to participate in the study to complete all surveys. Probands with a known molecular genetic diagnosis for their primary indication will be excluded. Additionally, probands are excluded if they have undergone a bone marrow transplant. Participants who have had clinical genetic results discussion that are non-diagnostic are withheld from enrollment until after 3 months from the time of discussion.

### Clinical genomic testing

TeleKidSeq participants will receive clinical genome sequencing from a Clinical Laboratory Improvement Amendments (CLIA)-certified and New York state-approved laboratory. Genome sequencing with mean coverage of at least 30× will be performed as singleton, duo, or trio sequencing depending on availability of biological parental samples. Probands and biological parents will have the option of receiving secondary findings, which include pathogenic and likely pathogenic variants in the 59 genes recommended by the American College of Medical Genetics and Genomics (ACMG) [27]. The ACMG v3.0 list for secondary findings policy statement was released after the study was underway and hence will not be adopted [28]. Sequencing analysis and variant classification will be performed based on the laboratory's individual variant interpretation pipeline, and Sanger validation of suspected pathogenic, likely pathogenic, or clinically suspicious variants will be performed.

### Study arms

Participants in the TeleKidSeq study will be randomized to one of two study arms. Participants in the ScrS arm will receive genome sequencing results by a study GC via Zoom videoconferencing platform with the use of its screen-sharing feature to display visual aids, including the patient’s personalized GUÍA report and genome sequencing report during the ROR session. Additional file 1 represents an example of a positive case visualized in GUÍA. For participants in the NScrS arm, results will be disclosed by a study GC via Zoom videoconferencing without the use of screen-sharing; however, a physical copy of the genome sequencing report and/or images approved by all TeleKidSeq GCs may be raised to the computer’s camera for the family to view. GCs will generate personalized GUÍA reports for all participants regardless of arm assignment. Following the ROR session, all participants will receive a PDF of their personalized GUÍA report along with a copy of the genome sequencing report either by secure email or by post, depending on the family’s preference.

## Procedures

**Fig. 2:**
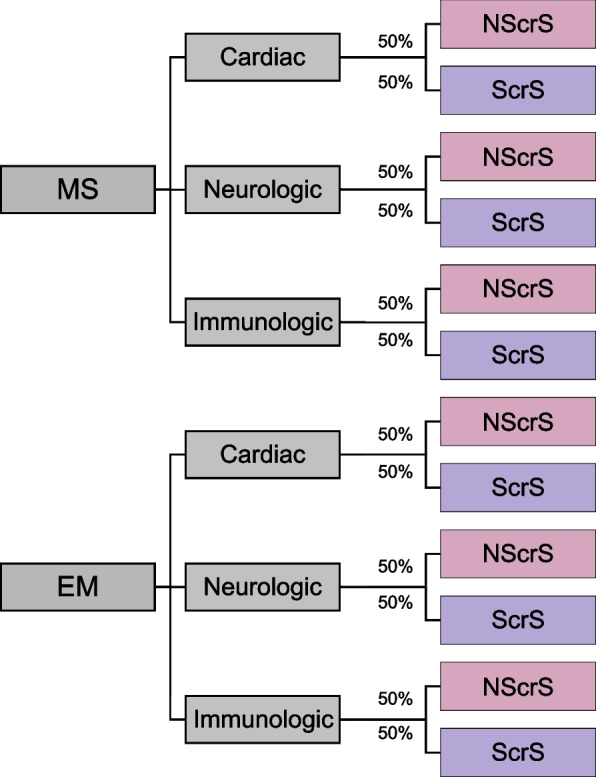
**Randomization schema.** The Icahn School of Medicine/Mount Sinai Health System (site 1) and the Albert Einstein College of Medicine/Montefiore Medical Center (site 2) are the two main sites for potential participants to be introduced to the study. Participants who are 0–21 years of age with an English- or Spanish-speaking parent or legal guardian are required to have a suspected underlying genetic cause that includes one of three main phenotypic categories: cardiology (cardiac), neurology (neurologic) or immunology (immunologic). For each category (cardiac, neurologic, and immunologic), half of the participants are randomized to the no screen-share arm (NScrS), and the other half are randomized to the screen-share arm (ScrS)

Figure 1 shows the study flow and data collection points of the TeleKidSeq pilot study. All participants will complete three study visits throughout their enrollment in the study. Participants will be randomized using a stratified randomization scheme by disease category (cardiac, neurologic, immunologic) and clinical site as seen in Fig. 2. Participants will be randomized to either the ScrS or NScrS study arm prior to the BL visit via a randomization module in REDCap. The REDCap random allocation mechanism will not be revealed to study staff at any point in the study.

### Baseline visit/electronic consent

Prior to the BL visit, the study team will ship the appropriate number of saliva kits with return packaging and postage to participants who are able to provide a saliva sample. For children who are under 6 years of age, incapable of providing a saliva sample, or whose previously submitted saliva sample has failed, a blood sample will be collected at a participating site. Parents/guardians of the proband will meet initially with the study staff via secure teleconferencing platform to complete the BL survey consent using an electronic consent form built into REDCap. The link to this form will be sent to the family via email or through the chat option in Zoom. Surveys will be conducted in either English or Spanish, depending on the participant’s preference.

Following the BL survey, all participants will undergo a standard pre-test genetic counseling encounter via videoconferencing. Pre-test genetic counseling involves educating the family on: the purpose of the study; describing the risks, benefits, and limitations of genome sequencing; reviewing possible types of results and the option to receive or decline secondary findings; discussing potential implications for other family members; and explaining data sharing options. Additionally, the GC will obtain a medical and family history. Informed consent for genome sequencing will be obtained using the electronic consent platform and a certified Spanish interpreter, as needed. After the BL visit, copies of the electronically signed consents (one pertaining to participation in surveys and one pertaining to genome sequencing) will be sent to the participant through email. For participants providing saliva samples, the study GC will review the sample collection process during the BL visit to ensure proper collection. Study enrollment will be contingent upon sample receipt by the laboratory. If one or more biological parents are not available during the BL visit, a saliva kit will be mailed to the parent’s home address. Parental consent and sample collection must be obtained within 2 weeks of the BL visit, if participating. BL visits will be conducted in the same manner across both study arms.

### Return of results/post-result surveys

Genome sequencing results will be reviewed by the GC, who interprets the genomic findings based on clinical interpretation guidelines developed by the study team. An ad hoc interpretation committee consisting of medical geneticists and a pediatric cardiologist with genetics expertise will review complex cases and aid with final diagnostic determinations and follow-up care recommendations. The genome sequencing results, clinical interpretation, and corresponding medical recommendations will be shared with the referring provider prior to disclosure of genomic results to the family for review and approval.

Each family will have a one-on-one results disclosure visit with a GC via a secure videoconferencing platform either with or without screen sharing, depending on randomization. Post-test genetic counseling for all participants will include review of the genome sequencing process and possible genome sequencing results; education on the proband’s genetic findings; discussion of any associated medical recommendations; explanation of the inheritance pattern, if known, reproductive and medical implications for relatives, if any; and referral to additional specialists or support services, as appropriate. Families will be encouraged to request that their referring provider and/or a clinical geneticist order reanalysis of inconclusive results every 12 months, as information about genetic variants, the patient’s phenotype, and their family medical history can change over time. Immediately after the results disclosure, a study staff member administers the ROR1 survey with the participant by videoconferencing. In rare cases, the ROR1 survey may be administered by telephone within 24 hours of results disclosure. After completion of the ROR1 visit, a copy of the results, a PDF copy of GUÍA, and any additional resources are sent to the family via email or post and uploaded to the patient’s electronic medical record. Six months after results disclosure, the staff will administer a final ROR2 survey by phone or via a secure videoconferencing, in either English or Spanish. Survey data will be stored in the REDCap database.

## Study outcomes

### Survey measures and outcomes

As described above, participant outcomes will be assessed through three surveys administered at BL, ROR1, and ROR2. Harmonized survey measures created by the CSER consortium to facilitate compilation of data from all CSER projects into a single data set for further analysis will be included [29], along with novel measures developed for the NYCKidSeq and TeleKidSeq studies adapted from previous studies to assess the impact of telehealth in our study population [30–32] (Table 1). A consortium-level effort by the CSER Survey Measures and Outcomes working group is currently underway to validate these novel measures for use in assessing the impact of genome-scale sequencing in the clinical care of diverse populations [1, 29]. The work of CSER will serve to further validate these measures for the purposes of studying the impact of genome sequencing in diverse populations.

**Table 1:** TeleKidSeq participant outcomes by survey timepoints

Variable	Source^a^	BL^b^	ROR1^c^	ROR2^d^
**Understanding**				
*Perceived understanding of genomic testing results*	NYCKidSeq (novel); CSER (novel); CSER measure adapted from Psychological Adaptation to Genetic Information Scale (PAGIS) [33]	**–**	**X**	**X**
*Objective understanding of genomic testing results*	NYCKidSeq (novel)	**–**	**X**	**X**
*Understanding of medical follow-up and actionability*	Adapted from CSER (novel): Recommended Medical Actions and Follow Through on Recommendations Attributable to Genomic Testing (MRA)	**–**	**X**	**–**
**Attitudes**				
*Expectations of genetic testing*	Adapted from Patient Reported Utility (PrU) [34]; NYCKidSeq (novel)	**X**	**–**	**–**
*Comfort with technology*	Adapted from Computer-Email-Web (CEW) Fluency Scale [35]	**X**	**–**	**–**
*Satisfaction with results*	CSER (novel)	**–**	**X**	**–**
*Satisfaction with communication mode*	CSER (novel), adapted for TeleKidSeq	**–**	**X**	**X**
*Perceptions of Satisfaction and Usefulness of Telehealth Delivery*	Adapted from Telemedicine Satisfaction and Usefulness Questionnaire (TSUQ) [30]	**–**	**X**	**–**
*Patient assessment of communication*	CSER measure adapted from Patient Assessment of cancer Communication Experiences (PACE) [36, 37]	**–**	**X**	**–**
*Evaluation of telehealth communication*	TeleKidSeq (novel) adapted from Lobb et al. 2006 [31] and Sanderson et al. 2016 [32]	**–**	**X**	**–**
*Satisfaction with interpretation and perceived cultural concordance (Spanish speakers only)*	CSER (novel)	**–**	**X**	**–**
*Evaluation of provided patient resources*	NYCKidSeq/TeleKidSeq (novel)	**–**	**–**	**X**
**Perceived utility**				
*Patient reported utility*	CSER measure adapted from Patient Reported Utility (PrU) [34]	**–**	**X**	**X**
**Psychological impact**				
*Feelings about genomic testing results*	CSER measure adapted from Feelings About Genomic Testing Results (FACToR) [38]	**–**	**X**	**X**
*Decisional regret* *(for positive secondary findings only)*	Adapted from Decision Regret Scale [39]	**–**	**X**	**X**
**Behavioral impact**		**–**		
*Information seeking*	CSER (novel)	**–**	**X**	**X**
*Adherence to medical follow-up recommendations; patient-initiated actions attributable to genomic testing*	Adapted from CSER (novel): Recommended Medical Actions and Follow Through on Recommendations Attributable to Genomic Testing (MRA); Patient-Initiated Actions Attributable to Genomic Testing (PIA)	**–**	**–**	**X**
*Family communication*	CSER (novel)	**–**	**–**	**X**
**Social impact**				
*Access to care*	CSER measure adapted from Medicare Expenditure Panel Survey, Household Component (MEPS-HC) [40]	**X**	**–**	**–**
*Access to technology*	TeleKidSeq measure (novel) adapted from CSER’s “Access to care” measure	**X**	**–**	**–**
*Quality of life ascertainment (for child)*	Pediatric Quality of Life Inventory (PedsQL) Parent Proxy Generic Core [41]; Adapted from EuroQol-Visual Analog Scale (VAS) [42]	**X**	**–**	**X**
**Economic impact**				
*Cost utility*	Adapted from Hebert et al. 2008 [43] and Valuation of Informal Care Questionnaire (iVICQ) [44]	**X**	**–**	**X**
**Sociodemographic factors**			**–**	
*Health literacy; subjective numeracy*	CSER measure adapted from BRIEF Health Literacy Survey [45]; CSER measure adapted from Subjective Numeracy Scale (SNS-3) [46]	**X**	**–**	**–**
*History of receiving genetic testing*	NYCKidSeq (novel) adapted from Genetic testing to Understand and Address Renal Disease Disparities (GUARDD) study [47]	**X**	**–**	**–**
*Trust in health care system*	CSER measure adapted from Health Care System Distrust Scale [48]	**X**	**–**	**–**
*Insurance status of child*	CSER measure adapted from National Health and Nutrition Examination Survey (NHANES) [49]	**X**	**–**	**X**
*Child only: sex, grandparent(s) country of origin*	CSER measure adapted from GenIUSS [50], CSER (novel)	**X**	**–**	**–**
*Child and Parent: age, race/ethnicity, country of origin, zip code*	Date of birth, CSER measure adapted from US Census [51, 52], CSER (novel), Zip code	**X**	**–**	**–**
*Parent only: education level, language, income, household, marital status*	Education and language: CSER (novel) Income and household: CSER measure adapted from NHANES [49] Marital status: NYCKidSeq (novel)	**X**	**–**	**–**
*Coronavirus impact*	Adapted from Coronavirus Impact Scale [53]	**X**	**–**	**–**

^a^NYCKidSeq (novel) measures were developed for the RCT, but were also used in the TeleKidSeq pilot study [2]. TeleKidSeq measures were developed specifically for this pilot study. NYCKidSeq measures which were adapted for the TeleKidSeq study are indicated. CSER measures were developed by a collaborative group of CSER investigators, as outlined in Goddard et al. 2020 [29]

^b^*BL* = baseline survey

^c^*ROR1* = return of results, visit 1 survey

^d^*ROR2* = return of results, visit 2 survey

The primary outcome of the TeleKidSeq study will be participants’ perceived understanding of their genome sequencing results, with a comparison between the ScrS and NScrS arms. Secondary outcomes will include objective understanding of genome sequencing results; understanding of medical follow up recommendations and the actionability of genome sequencing results; adherence to medical follow up recommendations made based on genomic results; and satisfaction with and ease of use of the telehealth experience, compared across the two arms. If results of this pilot study indicate that there is a signal toward effectiveness of the intervention in terms of participants’ perceived understanding or our secondary outcomes of interest, our team will consider submitting a grant application for a full-scale randomized trial. Diagnostic yield of genome sequencing will be assessed.

Additional participant outcomes will focus on six domains: (1) participant attitudes toward genomic testing and telehealth; (2) perceived utility of genomic testing and telehealth; (3) psychological and (4) behavioral impact of genomic testing; (5) social impact of genomic testing and telehealth on participants; as well as (6) economic impact of genomic testing as defined by physician costs. Sociodemographic factors will be collected as well as measures assessing the impact of the COVID-19 pandemic on participants and their families. A telehealth screener survey will be administered prior to enrollment in the study to capture data on potential participants’ access to devices and connectivity required for telehealth. The type and frequency of technical difficulties experienced during telehealth visits will also be documented in the study record by study staff to identify real-world barriers to telehealth.

Surveys will be administered by a staff member to reduce the risk of participant dropout, and surveys will be conducted over a period of approximately 9 months to minimize survey frequency fatigue (Table 2). To encourage participation in the study, compensation for completion of the final ROR2 survey will be higher than that offered for the BL and ROR1 surveys. The TeleKidSeq study surveys will be adapted from the NYCKidSeq study, which obtained survey feedback from participants during its lead-in pilot phase [2]. We will also structure surveys such that the order of questions is reflective of the significance of these measures to our outcomes.

**Table 2:** Schedule of forms and procedures (adapted from original SPIRIT table)^a^

	Study period							
Time point		− 3–0 months		BL (0 months)			ROR1 (3 months)	ROR2 (9 months)
Activity/assessment	Staff member	Referral/ eligibility screening	Randomization	Baseline survey	Pre-test genetic counseling	Sample collection	Results disclosure	6-month follow-up
Receipt of referral	Study coordinator	X						
Pre-screening of referral	Study coordinator/ genetic counselor	X						
Screening and scheduling of baseline visit	Study coordinator	X						
Randomization	Study coordinator		X					
Baseline survey informed consent	Study coordinator			X				
Administration of baseline survey	Study coordinator			X				
Pre-test genetic counseling	Genetic counselor				X			
Main study informed consent	Genetic counselor				X			
Sample collection for GS	Study phlebotomist/participants at home					X		
Receipt of results/genetic counselor preparation for ROR1	Genetic counselor						X	
Disclosure of results (ROR1)	Genetic counselor						X	
Administration of ROR1 survey	Study coordinator						X	
Administration of ROR2 survey	Study coordinator							X

^a^Recommended content can be displayed using various schematic formats. See SPIRIT 2013 Explanation and Elaboration for examples from protocols

### Analysis of outcomes

Quantitative survey measures will be reported using descriptive statistics. With the exception of economic impact, all outcomes will be compared between the two study arms. We will adjust for covariates such as race/ethnicity, age, and sex when appropriate. A mean score will be calculated for data collected using measures with summary scores, allowing us to account for missing data. Repeated chi-squared tests, regression models, and/or measures of analysis of variance (ANOVAs) will be fit to the data in a simple paired design to assess and identify significant improvements in participant understanding, satisfaction, and feelings about genome sequencing results, and result-related behaviors in the two study arms. A statistical significance criterion of *p* < 0.05 (after adjustment for multiple testing) will be used for all analyses.

The diagnostic yield will be calculated for the genome sequencing overall, by disease category (neurologic, cardiac, and immunologic), and by race/ethnic group. The diagnostic yield is assessed as the percentage of participants with definitive or likely positive diagnoses. The economic impact of genome sequencing results will be assessed by multivariable generalized linear models with the purpose of evaluating whether primary diagnostic result categories (positive/likely positive vs. uncertain vs. negative) are clinically informative and thus prompt downstream care activities and costs differentially. Differences in mean utilization rates and costs across result categories using a more meaningful linear scale (‘marginal means’) can be estimated by switching test categories to each possible level while keeping values of remaining covariables at the original value for each subject [54]. We will estimate 95% uncertainty intervals by bootstrapping.

As the primary purpose of this pilot study is to provide preliminary evidence of the efficacy of the intervention, we will also focus on descriptive statistics and more lenient thresholds of statistical significance. In addition to standard significance thresholds (alpha >= 0.05) or confidence intervals (> 95%), we will consider less stringent significance levels (i.e., alpha >= 0.1) or confidence intervals (> 85%) as suggestive evidence. Effect estimations and confidence intervals will be used to infer the size and direction of intervention effect to determine if there is evidence of a clinically important difference between the arms and to inform a decision whether to conduct a larger confirmatory trial.

## Confidentiality

As previously described [2], GCs and other study clinicians will access the participants’ medical records in accordance with the Health Insurance Portability and Accountability Act (HIPAA). Data will be stored and processed in a centralized location, with hard copies stored in locked files when not in use. Direct participant identifiers will be removed from data if not necessary for participant tracking. Participants may opt out of sharing de-identified genetic and related clinical information with external investigators and access-restricted scientific databases.

Regulatory documentation, research records, and remaining clinical research records will be retained for the amount of time required by the participating institutions. Documents will be stored and disposed of in accordance with sponsor and hospital requirements.

As communication with participants for the TeleKidSeq study primarily occurs electronically, additional measures will be taken to protect the storage and security of electronic data. Any email correspondence between the research teams will be secured using institutionally approved encryption and identifiable patient information is limited to the minimum necessary in order to uphold protection of patient privacy. The Zoom videoconferencing platform, which will be used for all videoconferencing visits, is configured to be HIPAA-compliant by both the Icahn School of Medicine at Mount Sinai and the Albert Einstein College of Medicine/Montefiore Medical Center.

## Discussion

The TeleKidSeq pilot study will explore the impact of real-time screen-sharing via videoconferencing platform in communicating genome sequencing results to the families of 496 participants with suspected genetic disorders. We will study participant outcomes including understanding, satisfaction, behaviors, and psychological and socioeconomic impact among participants from underrepresented groups who receive care in the New York metropolitan area. TeleKidSeq evolved out of the NYCKidSeq program to adapt to the changing landscape of healthcare service delivery during the COVID-19 pandemic. By randomizing participants to either the ScrS versus NScrS arm for results disclosure, we intend to capture the impact of shared visual aids in the comprehension and retention of discussed results. Additionally, we hope to identify barriers to videoconferencing with and without screen-sharing capabilities among patients underrepresented in genomic medicine research to help improve diverse families’ experiences with remote genetics services in the future.

Although remote genetic counseling studies to date have found that up to 37% of patients who have undergone a virtual counseling session prefer in-person visits to remote visits [55], we anticipate that this preference will decrease given the widespread acceptance of the use of virtual platforms for meetings and events. Studies that have examined the rapid transition to telehealth during the pandemic suggest that a hybrid model incorporating options for in-person and virtual visits may become the standard for genetic counseling practice moving forward [16]. In a survey by Dratch et al. of adult neurology patients’ preferences for service models in the midst of COVID-19, approximately 73% of participants preferred a hybrid model, whereas 25% preferred telehealth visits for future appointments [10]. This trend has also been observed among genetics providers [14]. Despite obstacles with sample collection, billing, and consenting, the majority of GCs hoped to continue using telehealth in genetics practice post pandemic. The outcomes of our study will help inform standardized approaches to remote genetics service delivery.

Limitations of TeleKidSeq include a lack of blinding participants’ randomization status to the participants, which may impose bias on these parties’ responses to outcome measures. Currently, GUÍA, the digital application used in our study for results disclosure, is only offered in English and Spanish. Additionally, GUÍA was developed before the COVID-19 pandemic to be used as visual communication aid for in-person visits, and therefore aspects such as font size may pose a challenge for participants in the ScrS arm who use mobile phones to receive results. Similar to most healthcare providers and parents during the pandemic, study personnel and families began using telehealth without extensive training. Families who were uncomfortable with videoconferencing technology or who did not have access to a device with videoconferencing capabilities are provided with study devices on hospital campuses for their visits. These participants’ experiences with telehealth could therefore be impacted by travel time and expenses as well as concern for COVID-19 exposure. Other variables to consider include the type and number of devices used during ROR, potential for respondent survey fatigue, the efficiency of Spanish interpretation, and the differences in dialects within the Spanish language.

TeleKidSeq aims to fill the gaps in current knowledge on the impact of visual aids in telehealth in diverse urban patient populations. As societal comfort with smart devices in healthcare steadily increases, with COVID-19 being a major catalyst, it will be important to recognize and examine the diversity of patients’ and providers’ experiences, preferences, and access barriers in using telehealth. By evaluating factors such as cost utility, psychological outcomes, and access to technology, we are generating evidence for best practices for genomic medicine implementation in health systems. These findings will also inform development of new digital tools to aid in conveying genomic concepts that will be made compatible with an ever-broadening array of smart devices and operating systems. In addition, this work will help address barriers and facilitate genomic medicine delivery approaches that are inclusive to diverse, multilingual populations.

## Supplementary Information

**Additional file 1.** An example of a positive result in GUÍA displayed in Spanish/English.

## Data Availability

De-identified data for this study will be shared in the NHGRI Genomic Data Science Analysis, Visualization, and Informatics Lab-space (AnVIL); a secure, access-restricted scientific research database. Interpretations of clinical significance of variants from genetic testing will be submitted to the ClinVar database at the National Institutes of Health. Data is shared based on participant data-sharing preferences.

## Abbreviations

BL: Baseline visit (study visit #1)
GC: Genetic counselor
GUÍA: Genomic Understanding, Information and Awareness application
NScrS: No screen-share
ROR: Return of results
ROR1: Return of results (study visit #2)
ROR2: Follow-up visit (study visit #3)
ScrS: Screen-share
