# Supplementary material for: The TeleKidSeq pilot study: incorporating telehealth into clinical care of children from diverse backgrounds undergoing whole genome sequencing
Source: Pilot Feasibility Stud. 2023 Mar 22;9:47. doi: 10.1186/s40814-023-01259-5 (PMC10031704; doi:10.1186/s40814-023-01259-5)
Supplement: Supplementary file 1 — Additional file 1. An example of a positive result in GUÍA displayed in Spanish/English. [file 40814_2023_1259_MOESM1_ESM.pdf]

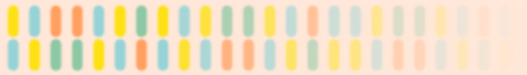

Resultados Genéticos de Jane

Jane's Genetic Test Results

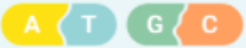

Aprende más sobre ADN

Learn About DNA

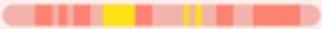

Aprende más sobre secuenciación del ADN

Learn About DNA Sequencing

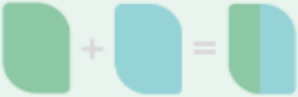

Familia

Family

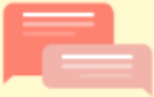

Recursos

Resources

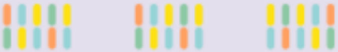

Descubrimientos Secundarios de Jane

Jane's Secondary Findings

Print All

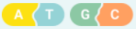

Aprende más sobre ADN

Una breve introducción a los fundamentos de la genética.

Learn About DNA

A short introduction to the basics of genetics.

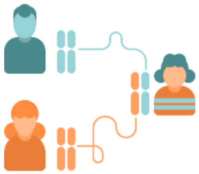

¿Qué son los genes?

Nuestros genes nos hacen quien somos. Los genes con los que nacemos se transmiten, o son heredados, de nuestra madre y nuestro padre. Cada persona tiene alrededor de 20,000 genes.

What are genes?

Our genes make us who we are. The genes we are born with are passed on, or inherited, from our mother and father. Every person has about 20,000 genes.

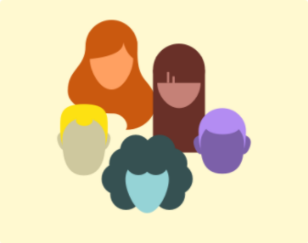

Nuestros genes controlan cómo nos vemos y cómo funciona nuestro cuerpo.

Our genes control what we look like and how our body works.

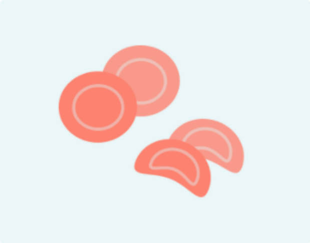

A veces, cuando un gen no funciona correctamente, este gen defectuoso puede causar problemas de salud.

Sometimes, when a gene is not working properly, this non-working gene can cause health problems.

Familia de Jane Jane's Family

Jane Family

Familia

¿Pueden los padres de Jane tener más hijos con Rubinstein-Taybi syndrome (RSTS)?

Evaluamos los padres de Jane para la variante genética de Jane. Como no descubrimos la variante genética de Jane en ninguno de sus padres, la madre y el padre de Jane tienen menos de un por ciento, o 1 en 100, de probabilidad de tener otro hijo juntos con Rubinstein-Taybi syndrome (RSTS). Si los padres de Jane están actualmente embarazados o están pensando en tener más hijos, sugerimos que vean a un consejero de genética reproductiva para revisar estos riesgos y hablar sobre cualquier opción reproductiva apropiada.

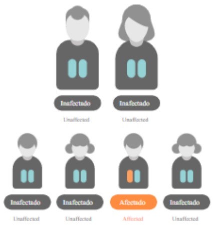

Can Jane's parents have more children with Rubinstein-Taybi syndrome (RSTS)?

We tested both Jane's parents for Jane's genetic variant. Since we did not find Jane's genetic variant in either of her parents, Jane's mother and father have a less than 1%, or 1 in 100, chance of having another child with Rubinstein-Taybi syndrome (RSTS) together. If Jane's parents are currently pregnant or thinking about having more children, we suggest that they see a prenatal genetic counselor to review these risks and discuss any appropriate prenatal options.
